# The Hemodynamically-Regulated Vascular Microenvironment Promotes Migration of the Steroidogenic Tissue during Its Interaction with Chromaffin Cells in the Zebrafish Embryo

**DOI:** 10.1371/journal.pone.0107997

**Published:** 2014-09-23

**Authors:** Chih-Wei Chou, You-Lin Zhuo, Zhe-Yu Jiang, Yi-Wen Liu

**Affiliations:** Department of Life Science, Tunghai University, Taichung, Taiwan; Institute of Cellular and Organismic Biology, Taiwan

## Abstract

**Background:**

While the endothelium-organ interaction is critical for regulating cellular behaviors during development and disease, the role of blood flow in these processes is only partially understood. The dorsal aorta performs paracrine functions for the timely migration and differentiation of the sympatho-adrenal system. However, it is unclear how the adrenal cortex and medulla achieve and maintain specific integration and whether hemodynamic forces play a role.

**Methodology and Principal Findings:**

In this study, the possible modulation of steroidogenic and chromaffin cell integration by blood flow was investigated in the teleostean counterpart of the adrenal gland, the interrenal gland, in the zebrafish (*Danio rerio*). Steroidogenic tissue migration and angiogenesis were suppressed by genetic or pharmacologic inhibition of blood flow, and enhanced by acceleration of blood flow upon norepinephrine treatment. Repressed steroidogenic tissue migration and angiogenesis due to flow deficiency were recoverable following restoration of flow. The regulation of interrenal morphogenesis by blood flow was found to be mediated through the vascular microenvironment and the Fibronectin-phosphorylated Focal Adhesion Kinase (Fn-pFak) signaling. Moreover, the knockdown of *krüppel-like factor 2a* (*klf2a*) or *matrix metalloproteinase 2* (*mmp2*), two genes regulated by the hemodynamic force, phenocopied the defects in migration, angiogenesis, the vascular microenvironment, and pFak signaling of the steroidogenic tissue observed in flow-deficient embryos, indicating a direct requirement of mechanotransduction in these processes. Interestingly, epithelial-type steroidogenic cells assumed a mesenchymal-like character and downregulated β-Catenin at cell-cell junctions during interaction with chromaffin cells, which was reversed by inhibiting blood flow or Fn-pFak signaling. Blood flow obstruction also affected the migration of chromaffin cells, but not through mechanosensitive or Fn-pFak dependent mechanisms.

**Conclusions and Significance:**

These results demonstrate that hemodynamically regulated Fn-pFak signaling promotes the migration of steroidogenic cells, ensuring their interaction with chromaffin cells along both sides of the midline during interrenal gland development.

## Introduction

Although blood vessels have long been known to respond to hemodynamic forces through mechanotransduction, only recently have researchers begun to understand the influence of hemodynamics on organogenesis through modulation of cellular behaviors, the extracellular matrix (ECM) microenvironment, as well as cell signaling events [1]. The early zebrafish embryo does not rely on blood circulation to transport oxygen [2], making it an excellent *in vivo* model for studying the effect of blood flow on development. Various genetic and pharmacological approaches have been developed in the zebrafish model, which have revealed the crucial role of hemodynamics in the morphogenesis of heart, kidney, and brain vasculature [3], [4], [5], [6]. Moreover, it is possible to study the role of hemodynamics in establishing the architecture of endocrine tissues in the zebrafish embryo, since the specification and differentiation of a variety of endocrine cells proceed even in the complete absence of vasculature [7], [8], [9], [10], [11].

How the adrenal cortex and medulla—arising because of distinct cell fate decisions in physically separated precursor cells—assemble to form the adrenal gland remains incompletely understood. The adrenal cortex is comprised of steroidogenic cells differentiated from the intermediate mesoderm, while the medulla contains chromaffin cells that originate from the neural crest and are subsequently segregated from the sympatho-adrenal lineage [12]. Mice deficient in the transcription factor steroidogenic factor-1 (SF-1, NR5A1) lack an adrenal cortex, but exhibit normal differentiation of chromaffin cells, half of which are present in the suprarenal region, arguing against a role for the adrenal cortex in attracting chromaffin cells [13]. However, ectopic adrenocortical cells in the mouse thorax, induced through the transgenic overexpression of SF-1, are capable of recruiting sympatho-adrenal progenitors [14]. These findings suggestive of an undefined role of the adrenal cortex have been clarified by the recent demonstration of the dorsal aorta (DA) as a morphogenetic center that instructs the specification and segregation of the sympatho-adrenal lineage in the chick embryo [15]. The DA and the adrenal cortex both secrete Neuregulin 1, which attracts chromaffin cells to the suprarenal region. However, the existence of shared paracrine factors does not explain why chromaffin cells colonize the adrenal cortex rather than non-adrenal regions surrounding the DA, and additional molecular and cellular factors could participate in the integration of steroidogenic and chromaffin cells. It was hypothesized that in addition to instructing the migration and differentiation of chromaffin cells, the vasculature near the adrenal gland also specifies the behavior of adrenocortical cells, thereby promoting cortex-medulla amalgamation. This possibility was investigated in the present study in zebrafish, an established model for exploring the development and diseases of the cardiovascular and endocrine systems.

The teleostean interrenal gland is functionally equivalent to the adrenal gland in mammals, with steroidogenic and chromaffin cell populations arising from conserved molecular programs [16], [17], [18]. The integration of these two cell types occurs between 1.5 and 3 days post-fertilization (dpf), which is immediately followed by *de novo* cortisol synthesis in response to stress [18], [19], [20]. Within the same temporal window, the interrenal vessel (IRV) is patterned along with a vessel-derived, Fibronectin (Fn)-enriched microenvironment [21], which is essential for IRV growth, steroidogenic tissue morphogenesis, and positioning the interrenal organ. Nevertheless, little is known about how the Fn-enriched interrenal microenvironment is regulated and the cellular mechanisms governing morphogenetic movements during integration.

Previous studies have shown that Klf2a and MMP2 are hemodynamically regulated: KLF2 is a transcription factor activated in cultured endothelial cells by fluid shear stress from laminar flow [22], [23], and the endothelial expression of mouse Klf2 and its ortholog *klf2a* in zebrafish reflects an increase in fluid-generated forces, while a loss of function leads to defective smooth muscle tone [24]. MMPs are known to mediate ECM remodeling and enable reshaping of tissues through peptidase activity [25]. In the zebrafish embryo, *mmp2* is expressed in the endothelium of developing axial vasculature in a flow-dependent manner [5]; and in rats and cultured cells, MMP2 activity in glomerular mesangial cells is induced by stretch [26], [27] and regulated by cyclic strains in the endothelium resulting from turbulent flow, which modulates the migration of vascular smooth muscle cells [28], [29]. Moreover, MMP2 cleaves a variety of ECM molecules, including type IV collagen, vitronectin, and fibronectin [30], [31]. Abundant RNA transcripts of both *klf2a* and *mmp2* are restrictively localized at the axial vasculature at around Prim-25 stage (36 hpf) during zebrafish development [5], [32], which is temporally correlated with the initiation of interrenal medial extension and angiogenesis. Furthermore, the nascent DA in the zebrafish does not recruit mural cells until 3 dpf [33], and differentiated vascular smooth muscle cells appear only after 7 dpf [34]; it was therefore hypothesized that hemodynamic forces could be transduced through the endothelium to influence closely associated interrenal cells.

In this study, the possible role of blood flow for the integration of steroidogenic and chromaffin cells was examined in the zebrafish interrenal gland, by using genetic and pharmacological approaches to abolish blood flow in the embryo. The vascular structure and associated ECM microenvironment in the interrenal region were examined for changes in the architecture of the developing interrenal tissue. The modulation of interrenal morphogenesis by blood flow through mechanotransduction was investigated by knocking down the mechanosensitive proteins Krüppel-like factor 2a (Klf2a) and Matrix metalloproteinase (Mmp)2. In addition, we demonstrated that steroidogenic cells undergo an epithelial-to-mesenchymal transition (EMT)-like change during organ assembly, which was correlated with a reduction in epithelial and a rise in mesenchymal markers. During EMT, which occurs at many critical steps during embryonic development, cell-cell contacts and polarity are lost and the cytoskeleton is extensively remodeled [35]. The present findings underscore the role of hemodynamics in regulating Fn-phosphorylated Focal adhesion kinase (pFak) signaling in the developing interrenal tissue, which in turn induces an EMT-like transformation in steroidogenic cells. Thus, in addition to the known chemoattractive function for chromaffin cells, the axial vasculature regulates the migration of steroidogenic cells through hemodynamically regulated signaling.

## Methods

### Ethics Statement

All of the zebrafish-use protocols in this research were reviewed and approved by the Institutional Animal Care and Use Committee of Tunghai University (IRB Approval NO. 101–12).

### Zebrafish Husbandry

Zebrafish (*Danio rerio*) were reared according to standard protocols [36]. Embryos were obtained from natural crosses of wild-type or transgenic fish, and staged as previously described [37]. The following lines were used: *Tg(wt1b: GFP)(line 1)* [38] (a gift from Christoph Englert, Fritz-Lipmann Institute, Jena, Germany); *Tg(ff1bEx2: GFP)* [39] (a gift from Dr. Woon-Khiong Chan, National University of Singapore); and *Tg(kdrl: EGFP)^s843^* [40] (a gift of Didier Stainier, University of California, San Francisco, CA, USA).

### 3β-Hydroxysteroid Dehydrogenase (3β-Hsd) Staining, In Situ Hybridization (ISH), Immunohistochemistry (IHC), Densitometry and Imaging

Embryos used for histological analysis were treated with 0.03% phenylthiourea (Sigma) from 12 h post-fertilization (hpf) onwards to inhibit pigmentation. The 3β-Hsd activity staining, ISH [9], and IHC [41] were performed with modifications according to previously published methods.

To delineate the morphology of steroidogenic interrenal tissue, histochemical staining for 3β-Hsd enzymatic activity was performed on whole embryos, and Nomarski images were captured using a BX51 microscope (Olympus).

For whole-mount ISH, digoxigenin (DIG)- and fluorescein-labeled antisense riboprobes were synthesized from linearized plasmids of *dopamine β hydroxylase* (*dβh*) and *ff1b* (*nr5a1a*) genes, respectively; the probes were detected with alkaline phosphatase-conjugated anti-DIG or -fluorescein antibody (Roche), and visualized with 5-bromo-4-chloro-3-indolyl-phosphate/nitro blue tetrazolium (Promega) or Fast Red (Roche). Stained embryos were flat-mounted and photographed under an Axioplan II microscope (Zeiss).

For IHC experiments, *Tg(ff1bEx2: GFP)* and *Tg(kdrl: EGFP)^s843^* embryos were fixed and embedded in 4% NuSieve GTG low-melting agarose (Lonza), cut into 100- µm sections with a VT1000M vibratome (Leica), and permeabilized with phosphate-buffered saline (PBS) containing 1% Triton X-100 before incubation with rabbit anti-human Fn (Sigma), mouse anti-human pFak (pY397) (BD Transduction Laboratories), mouse anti-chicken β-Catenin (Sigma), mouse anti-pig Vimentin (V9) (Abcam), and rabbit anti-zebrafish E-cadherin (Cdh1) (GeneTex) antibodies at 1∶200, 1∶100, 1∶50, 1∶200 and 1∶200 dilutions, respectively. Dylight 594- and 650-conjugated anti-rabbit or anti-mouse IgG (abcam) were used as secondary antibodies at 1∶200 dilution. Images were captured with an LSM510 confocal microscope with version 3.5 software (Zeiss).

For the quantification of 3β-Hsd activity, images of deyolked embryos in each group were taken with identical illumination and magnification using Axioskop 2 Plus microscope equipped with AxioVision 3.0 software (Carl Zeiss). Signal area and density were measured using Image Gauge Program, version 4.0 (Fuji Photo Film). Videos of embryos oriented with the anterior toward the left were taken by using an SMZ1500 microscope equipped with an AM4023X Dino-Eye eyepiece camera (Nikon).

### Microinjection of Antisense Morpholino Oligonucleotides (MOs)

The MO for *cardiac troponin T2a* (*tnnt2a*MO) (5′-CAT GTT TGC TCT GAT CTG ACA CGC A-3′) [42], along with *klf2a*MO (5′-GGA CCT GTC CAG TTC ATC CTT CCA C-3′) [43], and *mmp2*MO (5′-GGG AGC TTA GTA AAC ACA AAC CTG T-3′) [44] were synthesized by Genetools LLC and diluted in 1× Danieau solution, before injection into one- to two-cell stage embryos using a Nanoject (Drummond Scientific Company) at dosages of 1.0, 1.2, and 1.2 pmole per embryo, respectively.

### Pharmacological Treatment

Camptothecin (Sigma # C9911) treatment was performed according to a previously described method [45] with modifications. The compound (60 µM in 0.1% dimethyl sulfoxide [DMSO]) was applied to 48 hpf embryos, which were harvested at 57 hpf for the 3β-Hsd activity assay. The treatment of embryos with 2,3-butanedione 2-monoxime (2,3-BDM; Sigma #B0753) was as described in an earlier report [46], except that dechorionated embryos were immersed in various concentrations of 2,3-BDM starting from 1.5 dpf. Norepinephrine treatment was performed by treating dechorionated embryos with 0.01, 0.1 or 1 mM norepinephrine (Sigma A7257) freshly prepared in egg water. L-NAME treatment was performed by treating dechorionated embryos with freshly prepared 100 µM Nω-nitro-l-arginine methyl ester (l-NAME) (#N5751, Sigma) in egg water with 0.1% DMSO at 36 hpf. For l-arginyl-l-glycyl-l-aspartic acid (RGD; #G1269, Sigma) treatment, the peptide was reconstituted to 1 mM in filter-sterilized egg water and applied to dechorionated embryos at a final concentration of 100 µM at 26 hpf; embryos were collected at 2.5 dpf and fixed for histological assays.

### Statistical Analysis

All quantitative data are expressed as the mean ± standard error of the mean. Data were evaluated by analysis of variance (ANOVA), followed by Duncan's new multiple range test (Duncan's multiple test) or Student's t test. *P*<0.05 was considered statistically significant.

## Results

### Blood Flow is Required for the Morphogenesis of Kidney Glomerulus and Interrenal Tissue

**Figure 1:**
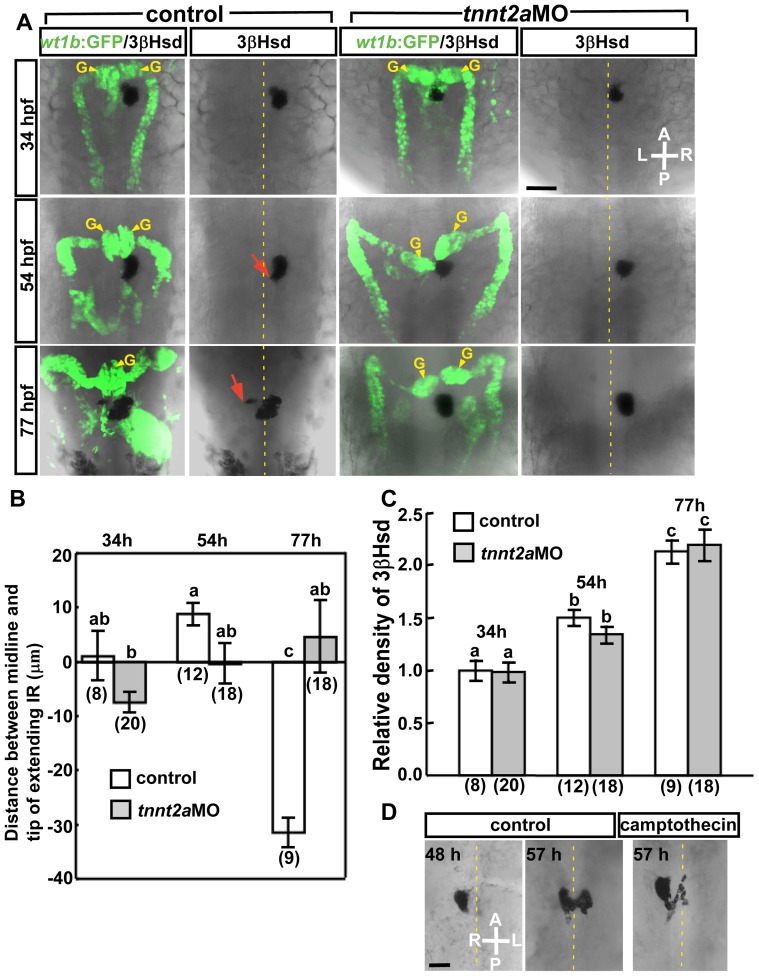
Morphology of pronephros and interrenal tissue in the absence of blood flow. (A) The interrenal steroidogenic tissue positive for 3β-Hsd activity forms an extension that protrudes toward the midline by 54 hpf (cell protrusions marked by red arrows), while kidney glomeruli delineated by *wt1b*: GFP expression (G, yellow arrowheads) assemble at the midline. Morphogenetic movements of kidney glomeruli and steroidogenic tissues are defective in the *tnnt2a* morphant. All panels show dorsal views of representative embryos. (B) Quantification of effects of *tnnt2a*MO injection on interrenal migration. The distance between the midline and the migrating tip of steroidogenic tissue was designated as positive if the migrating tip had not reached the midline, and negative if the tip had migrated across the midline. (C) Relative density of steroidogenic tissue, as assessed by 3β-Hsd activity staining in the ventral surface, in *tnnt2a* morphants compared to wild-type controls. The number of embryos in each group is indicated in parentheses in (B) and (C). Histograms with different letters above them are significantly different (ANOVA and Duncan's multiple test, *P<*0.05). (D) Effect of camptothecin treatment from 48 to 57 hpf on 3β-Hsd activity in steroidogenic cells. A, anterior; P, posterior; L, left; R, right. Broken yellow lines indicate position of the midline. Abbreviations: glomerulus (G). Scale bar, 50 µm.

The hemodynamic force drives the assembly of zebrafish kidney glomeruli at the midline [5]. Since the kidney and interrenal gland develop in parallel [16], with the DA acting as the source of angiogenesis in both organs [21], [47], the role of blood flow in the morphogenesis of the interrenal gland was assessed. Kidney and interrenal tissue morphology was visualized by staining for 3β-Hsd enzymatic activity in *Tg(wt1b: GFP)* embryos, in which GFP is expressed in the glomerular podocytes, pronephric tubules, and proximal pronephric ducts [38], [48], as well as in the exocrine pancreas due to a possible position effect of transgene insertion. The MO against *tnnt2a*, a gene essential for sarcomere assembly and heart contractility [42], was injected into *Tg(wt1b: GFP)* embryos, and 100% of the *tnnt2a* morphants (n = 56) displayed completely abolished heartbeat and blood flow. Consistent with the previous study [5], bilateral kidney glomeruli in control embryos assembled at the midline by 54 hpf, but failed to fuse in *tnnt2a* morphants (Figure 1A). The steroidogenic tissue in blood flow-deficient embryos grew as a round, tightly packed cell aggregate, without extending protrusions as in the case of controls. The extent of migration was quantified by measuring the distance between the tip of medially extending steroidogenic tissue and the midline, and although there was no difference at 54 hpf, the distance was decreased in morphants relative to control embryos at 77 hpf (Figure 1B). The inhibition of medial extension was not due to growth arrest of interrenal tissue, since organ size—as assessed by densitometric analysis of 3β-Hsd activity staining—was similar in *tnnt2a*MO-injected and control embryos at all stages examined (Figure 1C). To further evaluate the effect of tissue growth on interrenal morphogenetic movement, embryos were treated with camptothecin, which blocks cell proliferation in zebrafish embryos [45], from 48 to 57 hpf. Camptothecin-treated embryos showed an 18% reduction in interrenal tissue size compared to controls. Notably, the formation of protrusions was unaffected by camptothecin treatment (Figure 1D), suggesting that cell growth is not the major determinant for interrenal medial extension. Taken together, these results indicate that blood flow is required for the morphogenetic movement of both kidney and interrenal tissues. Based on the early defects in the ventral DA caused by *tnnt2a* knockdown [49], [50], experiments were performed to establish whether interrenal morphogenetic movement during the temporal window of organ assembly is specifically subject to regulation by blood flow.

### Medial Extension of Steroidogenic Tissue during Interrenal Organ Assembly is Regulated by Blood Flow

**Figure 2:**
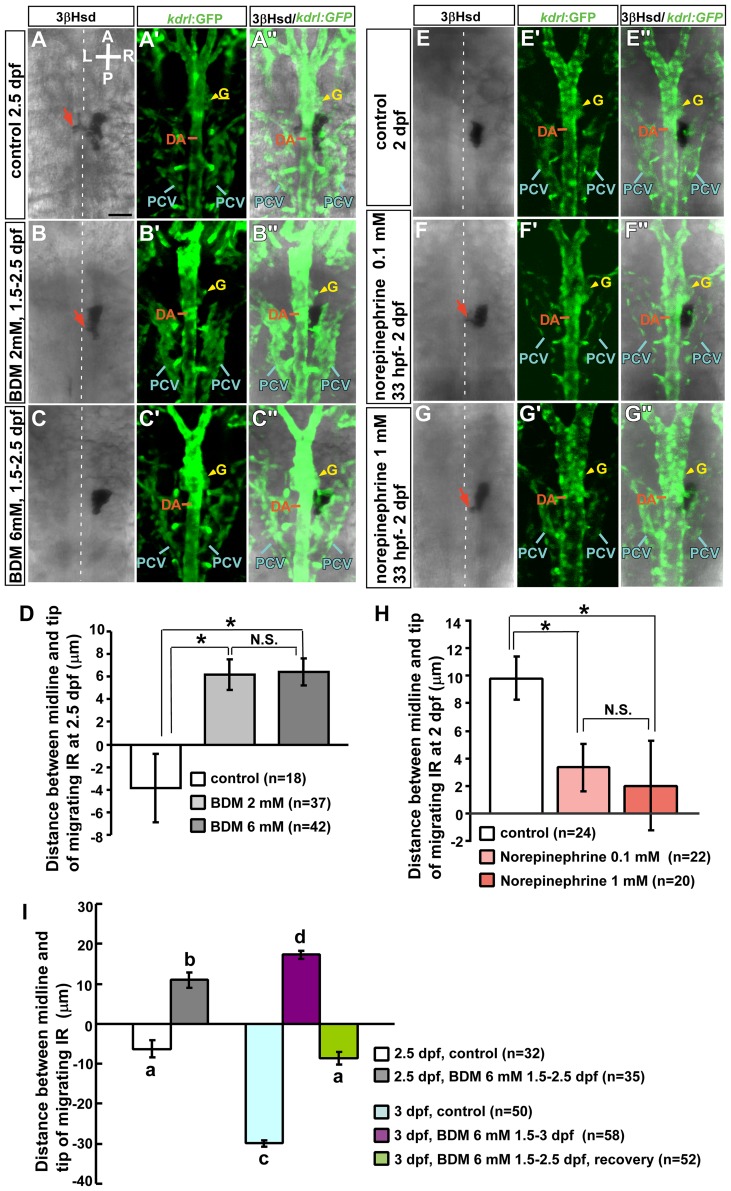
Effects of 2,3-BDM and norepinephrine on morphogenetic movements of interrenal tissue. For repression of blood flow, *Tg(kdrl: GFP)^s843^* embryos were treated with (A–A'') vehicle (control), or 2,3-BDM at a concentration of (B–B'') 2 mM or (C–C'') 6 mM from 1.5 dpf. A suppression of the medial extension of steroidogenic cells was observed in 2,3-BDM-treated embryos at 2.5 dpf, while the morphology of the DA and the pronephric glomerulus (yellow arrowheads) appeared unperturbed. Protrusions (red arrow) formed at the lower concentration; the phenotype was more severe at the higher concentration. For acceleration of blood flow, *Tg(kdrl: GFP)^s843^* embryos were treated with (E-E'') vehicle (control), or norepinephrine at a concentration of (F–F'') 0.1 mM or (G-G'') 1 mM from 33 hpf. An enhancement of interrenal medial extension, as evidenced by the formation of protrusions, was observed in norepinephrine-treated embryos at 2 dpf. The effects of 2,3-BDM and norepinephrine treatments on interrenal migration were quantified in (D) and (H), respectively. The distance between the midline and migrating tip of steroidogenic tissue was designated as positive if the migrating tip had not reached the midline, and negative if the tip had migrated across the midline. *P<0.05; N.S., not significant (Student's t-test). (I) Suppressing effect of interrenal cell migration by 2,3-BDM at 6 mM from 1.5 dpf was reversible at 3 dpf, as the 2,3-BDM applied from 1.5 dpf was washed out at 2.5 dpf for restoring blood flow. The interrenal tissue in recovered embryos extended across the midline at 3 dpf and displayed a migration distance not significantly different from that in control embryos at 2.5 dpf. Histograms with different letters above them are significantly different (ANOVA and Duncan's multiple test, *P<*0.05). A, anterior; P, posterior; L, left; R, right. Broken white lines indicate position of the midline. Abbreviations: glomerulus (G), posterior cardinal vein (PCV). Scale bar, 50 µm.

The pharmacological agent 2,3-BDM, which affects heart rate without affecting cell viability, has previously been used to evaluate the role of blood flow in zebrafish organogenesis [5], [6], [51]. To rule out the possibility that defective medial extension of the interrenal tissue in *tnnt2a* morphants was due to an early effect on blood vessel morphogenesis, blood flow was inhibited in embryos by application of 2,3-BDM from 1.5 dpf, by which interrenal medial extension and organ assembly are initiated (Figure 2). The 2,3-BDM treatment on zebrafish embryos leads to decreased myofibrillar ATPase and myocardial force in a dose-dependent manner, which affects heart rate at a concentration as low as 2 mM and is sufficient to eliminate blood flow at 6 mM [46]. Consistent with the previous study, a concentration of 6 mM produced a 53% reduction in heart rate and a cessation of blood flow (Video S1) compared to control embryos (Video S2). A lower concentration of 2,3-BDM (2 mM) caused a 26% decrease in heart rate and a visibly weakened blood flow (Video S3). To examine the morphology of the DA adjacent to the kidney and interrenal regions, 2,3-BDM was applied to *Tg(kdrl: EGFP)^s843^* embryos that express GFP in the developing blood vascular structure [40], which were then harvested at 2.5 dpf, when the migration of interrenal cells can be clearly observed [19]. Treatment with 2 or 6 mM 2,3-BDM suppressed interrenal medial extension across the midline (Figure 2B, B'', C, C''), with similar effects observed at both concentrations (Figure 2D). However, the interrenal tissue had more protrusions at 2 than at 6 mM 2,3-BDM (Figure 2B, C), suggesting that the effect of 2,3-BDM was dose-dependent. In contrast, the morphology of the DA and the pronephric glomerulus was unperturbed by 2,3-BDM treatment. Thus, the inhibition of medial extension of the interrenal tissue caused by loss of blood flow was not due to a general defect in the DA.

Conversely, as the heart rate was accelerated by norepinephrine treatment from 33 hpf, a significant enhancement of interrenal tissue extension was detected at 2 dpf (Figure 2E-E'', F-F'', G-G'', H). Norepinephrine accelerated the heart rate of developing embryos in a dose-dependent manner (Figure S1). 0.01, 0.1 and 1 mM of norepinephrine treatments on embryos at 33 hpf led to a 23%, 29% and 41% increase of heart rate, respectively. Compared to the control embryo (Figure 2E), both 0.1 and 1 mM of norepinephrine treatements led to a more evident migratory phenotype of interrenal tissue, as verified from the extending protrusions (red arrows in Figure 2F, G), which was consistent with the results of the quantification of interrenal tissue extension (Figure 2H). However, no significant difference in steroidogenic tissue extension could be detected between embryos treated with 0.1 or 1 mM of norepinephrine. Norepinephrine at 0.01 mM also enhanced steroidogenic tissue extension (4.3±2.0 µm, n = 24) compared to control embryos, while no significant statistical difference was found among norepinephrine treatments at 0.01, 0.1 and 1 mM. Our results thus indicated that a moderate elevation of heart rate by 23% was sufficient to promote migration of steroidogenic interrenal cells, although further increase of heart rate by treating with higher concentrations of norepinephrine did not lead to a dose-dependent enhancing effect on interrenal medial extension.

To confirm the relationship between blood flow and steroidogenic cell migration, we further tested whether interrenal medial extension repressed by 2,3-BDM treatment could be recovered by restoring the blood flow (Figure 2I, Figure S2). Steroidogenic tissue migrated across the midline by 2.5 dpf (Figure S2A, A'') and continued to extend and form a bilobed organ structure by 3 dpf (Figure S2C, C''). Extension of steroidogenic tissue was arrested as 2,3-BDM at 6 mM was applied to embryos from 1.5 dpf onwards (Figure S2B, B'', D, D''). The steroidogenic tissue extension in 2,3-BDM-treated embryos was recovered at 3 dpf as 2,3-BDM was washed out at 2.5 dpf (Figure S2E, E''). The interrenal tissue in 2,3-BDM-treated embryos appeared to be located further away from the midline at 3 dpf than at 2.5 dpf (Figure 2I), possibly due to continuous growth of peri-interrenal structures from 2.5 to 3 dpf. It is interesting to note that there was no significant difference of migration distance between control embryos at 2.5 dpf and recovered embryos at 3 dpf (Figure 2I), implying that the inhibited interrenal medial extension by 2,3-BDM treatment from 1.5 to 2.5 dpf could be rescued by resuming blood flow for 12 hours. Taken together, results in Figure 2 demonstrated that pharmacologic repression and acceleration of heart rates are well correlated with the extent of interrenal medial extension. Furthermore, the inhibited steroidogenic tissue extension caused by arrested blood flow was recoverable following restoration of blood flow, providing strong evidence that migratory activity of interrenal steroidogenic cells is indeed modulated by blood flow.

### Blood Flow Regulates IRV Extension during Interrenal Organ Assembly

**Figure 3:**
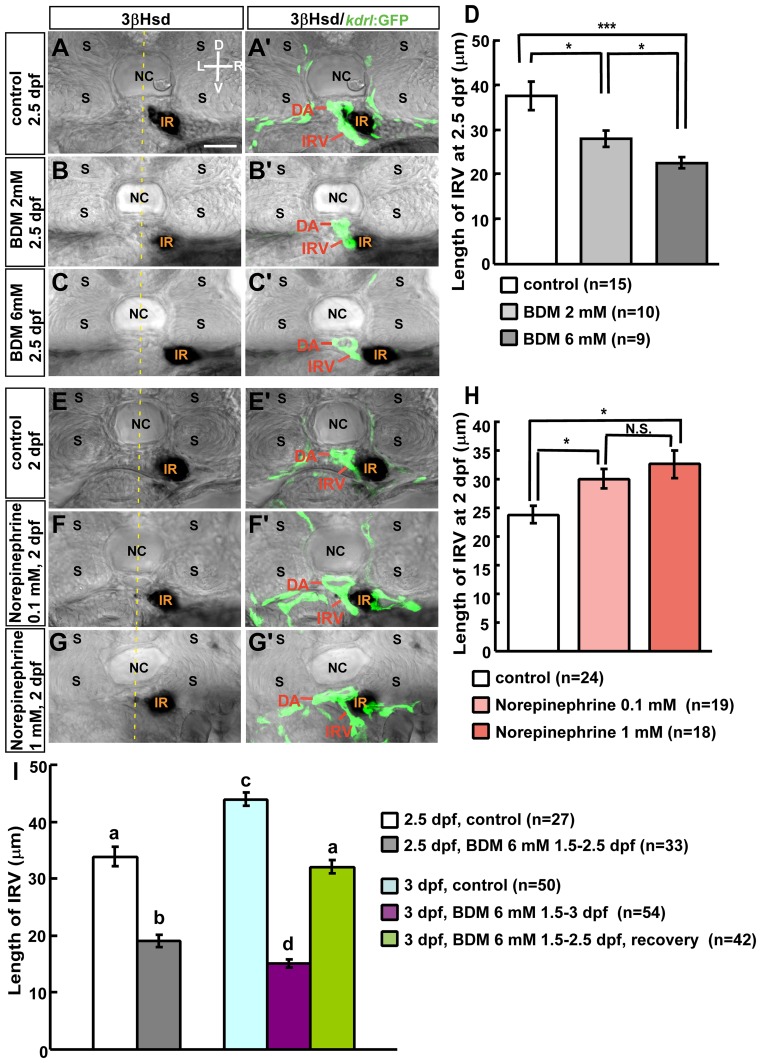
Effects of 2,3-BDM and norepinephrine on IRV formation. For repression of blood flow, *Tg(kdrl: GFP)^s843^* embryos were treated with (A–A') vehicle (control), or 2,3-BDM at a concentration of (B–B') 2 mM or (C–C') 6 mM from 1.5 dpf, and harvested at 2.5 dpf. For acceleration of blood flow, *Tg(kdrl: GFP)^s843^* embryos were treated with (E-E') vehicle (control), or norepinephrine at a concentration of (F–F') 0.1 mM or (G-G') 1 mM from 33 hpf, and harvested at 2 dpf. Transverse sections of harvested embryos were subject to analysis of 3β-Hsd activity (black) and GFP expression (green). IRV lengths of 2,3-BDM- or norepinephrine-treated embryos were quantified in (D) and (H), respectively; which were verified from confocal Z-stacks covering the full range of IRV growth, and measurements were made from single focal planes displaying the maximal range of ventrally extending IRV. *P<0.05, ***P<0.0005, N.S., not significant (Student's t-test). (I) Repressing effect of 2,3-BDM (6 mM) on IRV growth was reversible at 3 dpf, as the 2,3-BDM applied from 1.5 dpf was washed out at 2.5 dpf. The IRV length in recovered embryos at 3 dpf was not significantly different from that in control embryos at 2.5 dpf. Histograms with different letters above them are significantly different (ANOVA and Duncan's multiple test, *P<*0.05). D, dorsal; V, ventral; L, left; R, right. Abbreviations: interrenal tissue (IR), notochord (NC), somite (S). Scale bar, 25 µm.

Interrenal medial extension temporally coincides with IRV angiogenesis, which is promoted by the IRV-associated vascular microenvironment [21], and therefore the effect of reduced blood flow on IRV angiogenesis was examined from 1.5 to 2.5 dpf (Figure 3). IRV growth was initiated normally when blood flow was inhibited starting at 1.5 dpf. However, in embryos treated with 2 or 6 mM 2,3-BDM, IRV lengths were reduced and the vessels reached but did not extend ventrally through the interrenal tissue, with a more severe phenotype observed at the higher concentration (Figure 3B'–C', D), indicating a dose-dependent effect of 2,3-BDM on IRV extension. Our previous study showed that IRV directionality, but not initiation of angiogenesis, is perturbed in the *tnnt2a* morphant [21]; accordingly, the present results indicated that the blood flow was not required for the sprouting of the IRV from the DA, but may play a role in its extension. Furthermore, the interrenal tissue in 2,3-BDM-treated embryos (Figure 3B–B', C–C') and *tnnt2a* morphants [21] only interacted with the tip of the IRV but not the DA, while the extending interrenal tissue in the control embryo remained closely associated with both the ventral DA and the IRV (Figure 3A–A').

To test whether accelerated blood flow could promote extension of the IRV, embryos were treated with norepinephrine at 0.1 or 1 mM at 33 hpf and harvested at 2 dpf for analysis (Figure 3E-G, E'-G'). It was found that norepinephrine at both concentrations significantly increased length of the IRV (Figure 3H), yet no difference in the IRV growth was observed between 0.1 and 1 mM of norepinephrine treatments. The promoting effects of norepinephrine treatments on extension of the IRV were therefore highly correlated with those on interrenal medial extension (Figure 2H). Similar to the case of interrenal medial extension in Figure 2I, restoring blood flow by 2,3-BDM washout after the treatment from 1.5 to 2.5 dpf led to a recovery of IRV growth at 3 dpf, with the IRV length in 3-dpf recovered embryos not significantly different from that in 2.5-dpf control embryos (Figure 3I, S3).

Our results from pharmacologic inhibition and acceleration of blood flow therefore strongly support that blood flow regulates both interrenal medial extension and IRV growth during interrenal organ assembly. While the processes of interrenal medial extension and IRV growth occur synchronously during development [21], they are both influenced by blood flow in a highly correlated manner. Therefore, it leads to the hypothesis that there might be a common flow-regulated molecular and cellular mechanism by which both interrenal medial extension and IRV angiogenesis are regulated. The initiation of IRV angiogenesis requires deposition of Fn at the ventral DA near the interrenal tissue, and the accumulation of this protein in the local microenvironment supports IRV extension [21]. While the interrenal tissue is closely associated with both the DA and the IRV, vessel-derived Fn functions at the tissue-vessel interface and thus modulates the migration of steroidogenic cells [19]. Therefore, it is possible that the vascular microenvironment established during IRV angiogenesis is regulated by blood flow, which in turn modulates the migration of steroidogenic cells.

### Blood Flow is Required for Patterning the Fn-rich Microenvironment and pFak Distribution in the Interrenal Region

**Figure 4:**
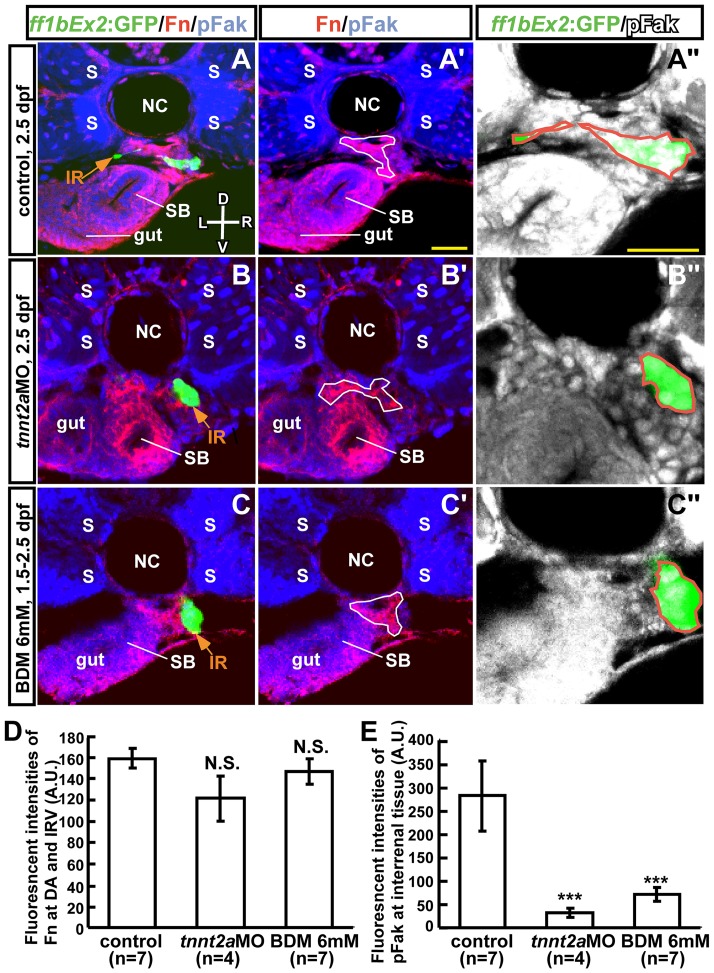
Effect of blood flow inhibition on the ECM microenvironment and pFak distribution in interrenal steroidogenic tissue. Transverse sections of *Tg(ff1bEx2: GFP)* embryos were (A–A'') uninjected (control), (B–B'') injected with *tnnt2a*MO, or (C–C'') treated with 6 mM 2,3-BDM from 1.5 dpf. Embryos were harvested at 2.5 dpf and assayed for expression of GFP (green), Fn (red), and pFak (blue in A–C and A'–C'; white in A''–C''). Images are single confocal planes showing the maximal transverse dimension of *ff1b*GFP-expressing steroidogenic tissue of a representative embryo, with magnified views shown in (A''–C''). (D) Fluorescence intensity of Fn in the DA and IRV selected as regions of interest (ROI; white lines in A'–C') normalized to the size of the ROI. (E) Total fluorescence intensities of pFak within the steroidogenic tissue (ROI marked by orange lines in A''–C'') were normalized to the size of the cluster. The difference between the treatment and the control groups was analyzed by Student's t-test. ***P<0.001, N.S., not significant. D, dorsal; V, ventral; L, left; R, right. Abbreviations: arbitrary units (A.U.), interrenal tissue (IR), notochord (NC), somite (S), swim bladder (SB). Scale bar, 25 µm.

Possible perturbations in the interrenal microenvironment of blood flow-deficient embryos were assessed using the *Tg(ff1bEx2: GFP)* line, in which the GFP expression recapitulates the endogenous expression of *ff1b* [52]—the teleostean ortholog of mammalian SF-1—and marks the ontogeny of steroidogenic interrenal tissue [16], [17]. Embryos were examined for expression of Fn and pFak (Figure 4). The elimination of blood flow by *tnnt2a*MO injection (Figure 4B–B'') or 6 mM 2,3-BDM treatment (Figure 4C–C'') did not diminish Fn accumulation in the interrenal microenvironment (Figure 4D). However, Fn was abnormally distributed, raising the possibility that in the absence of blood flow, the polymerization of Fn into fibrils was disrupted. To verify whether aberrant Fn deposition perturbed signaling events within the interrenal tissue, the localization of pFak—a downstream effector of Fn-Integrin signaling and an indicator of the dynamic reorganization of focal adhesions during cell migration—was examined (Figure 4A–C''). Indeed, pFak level within the interrenal tissue was significantly reduced compared to control embryos (Figure 4E), suggesting a disruption of Integrin-mediated signaling. In contrast, pFak distribution was readily detected at the somites, gut tube, and swim bladder in *tnnt2a* morphants (Figure 4B, B') and 2,3-BDM-treated embryos (Figure 4C, C'). These results indicate that the interrenal microenvironment established during IRV angiogenesis is perturbed by reduced blood flow, resulting in the suppression of Fn-pFak signaling and steroidogenic cell migration.

### Klf2a and MMP2 are Required for Migration, Angiogenesis, and Fn-pFak signaling in the interrenal tissue

**Figure 5:**
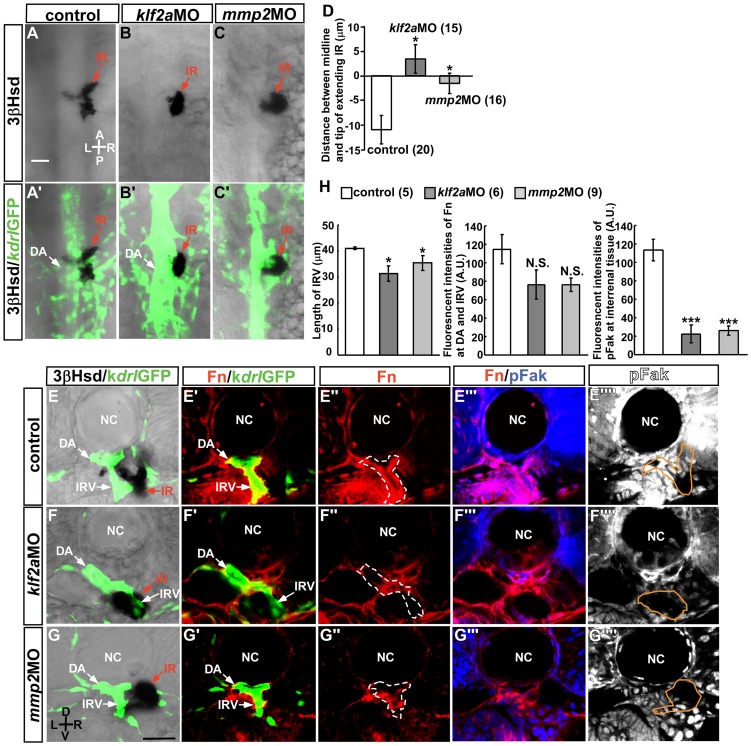
Suppression of interrenal tissue migration in *klf2a* and *mmp2* morphants. Dorsal view of interrenal steroidogenic tissue (IR, red arrows) as detected by 3β-Hsd activity staining, with adjacent vasculature marked by GFP expression. *Tg(kdrl: GFP)^s843^* embryos were (A, A') uninjected (control), or injected with (B, B') *klf2a*MO or (C, C') *mmp2*MO. (D) Quantification of the effects of MO-mediated gene knockdown on interrenal migration. The distance between the midline and the migrating tip of steroidogenic tissue was designated as positive if the migrating tip had not reached the midline and negative if it had crossed the midline. The number of embryos in each group is indicated in parentheses. The extent of interrenal medial extension of control 2.5-dpf embryos in panel 5D was not statistically different from those in Figure 2D and 2I. Fn and pFak expression in the interrenal region was examined in (E–E'''') uninjected (control), and (F–F'''') *klf2a*MO- and (G–G'''') *mmp2*MO-injected embryos by IHC. Images show transverse sections of a representative embryo from each treatment group. (H) Quantification of the effects of *klf2a*MO and *mmp2*MO on IRV growth, Fn level in the vicinity of the DA and IRV (ROI marked by broken lines in E''–G''), and pFak level in the steroidogenic tissue (ROI marked by orange lines in E''''–G''''). The number of embryos in each group is indicated in parentheses. Fluorescence intensities of Fn and pFak were normalized to their respective ROI sizes. The difference between the treatment and the control groups was analyzed by Student's t-test. *P<0.05, ***P<0.001, N.S., not significant. A, anterior; P, posterior; L, left; R, right; D, dorsal; V, ventral. Abbreviations: notochord (NC). Scale bar, 25 µm.

To confirm whether hemodynamics, and not circulating factors, account for the effect of blood flow on steroidogenic tissue migration and angiogenesis, the role of Klf2a and MMP2 in interrenal morphogenesis was evaluated (Figure 5). While *Klf2a* deficiency leads to heart failure at 3 dpf [24], as evidenced by pericardial edema and venous pooling of blood around the yolk sac, a heart rate similar to that of control embryos is observed at 2 dpf [3]. In the present study, only mild cardiac edema was observed at 2.5 dpf, and circulation was unaffected, making it possible to evaluate the specific effect of hemodynamic forces on interrenal development at this stage. Consistent with the previous finding that primary vascular structures are not perturbed in the *klf2a* morphant [24], the axial vasculature was grossly normal in the peri-interrenal region at 2.5 dpf (Figure 5B'). However, the migration of steroidogenic cells was inhibited (Figure 5B, D), and IRV growth and directionality were perturbed (Figure 5F, H). In contrast to the *tnnt2a* morphant and 2,3-BDM-treated embryos in which the steroidogenic tissue and DA were not closely associated (Figure 3) [21], the interrenal tissue was proximal to the DA in the *klf2a* morphant. Despite these phenotypic differences between blood flow-deficient and *klf2a* morphant embryos, both types of embryos showed abnormal Fn distribution and reduced pFak level in the interrenal area (Figure 5F'–F'''', H).

Although blood flow regulates the generation of hematopoietic stem cells from the DA by a *klf2a*-*nitric oxide* (NO) pathway [49], [50], steroidogenic interrenal tissue migration and angiogenesis were NO-independent (Figure S4). Treatment with the endothelial NO synthase inhibitor l-NAME from 1.5 to 2.5 dpf had no effect on interrenal tissue migration and angiogenesis, indicating that blood flow and Klf2a do not regulate interrenal morphogenesis through activation of NO signaling.

Embryos injected with 1.2 pmole *mmp2*MO had no major morphological abnormalities except for a kinked tail and mild (2%) reduction in heartbeat that did not cause any visible changes in blood flow. Nevertheless, morphants showed defects in migration, angiogenesis, and Fn-pFak signaling in the interrenal tissue (Figure 5C, D, G, H). This indicated that MMP2 activity may participate in the blood flow-regulated interrenal microenvironment. As observed upon *klf2a* knockdown, the steroidogenic tissue remained associated with both the DA and the IRV, suggesting that neither Klf2a nor MMP2 was essential for this association, which likely depends on other hemodynamically regulated molecules.

### Medial Extension of the Interrenal Tissue Involves EMT-like Changes in Steroidogenic Cell Morphology that are Hemodynamically Regulated and pFak-Dependent

**Figure 6:**
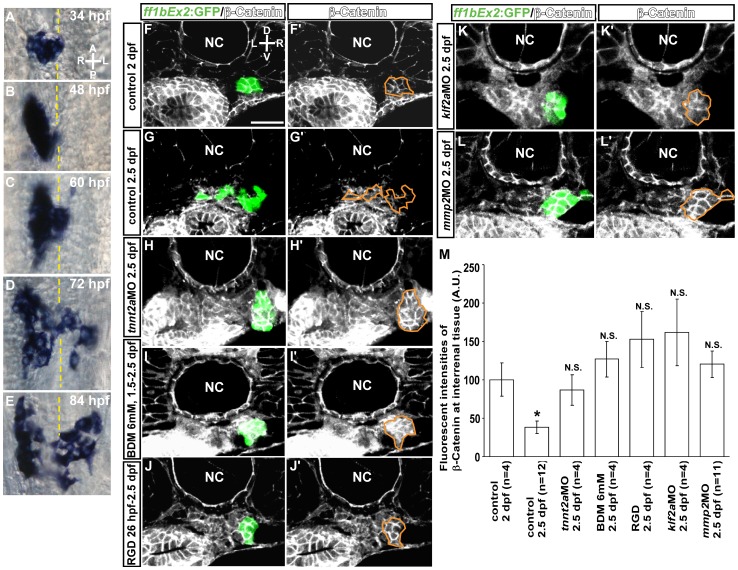
Steroidogenic cells are induced to undergo an EMT-like change by hemodynamic forces and pFak signaling. (A–E) Ventral view of the midtrunk from 34 to 84 hpf; steroidogenic cells become loosely associated and develop protrusions at the cell surface. The accumulation of β-Catenin at cell-cell junctions in the steroidogenic tissue can be seen in cross sections of *Tg(ff1bEx2: GFP)* embryos at (F, F') 2 dpf, but not at (G, G') 2.5 dpf. The decrease in junctional β-Catenin was not observed in (H, H') *tnnt2a* morphants, (I, I') 2,3-BDM- or (J, J') RGD-treated embryos, or (K, K') *klf2a* or (L, L') *mmp2* morphants. Sections are shown of a representative embryo from each treatment group. (M) Fluorescence intensity of β-Catenin in *ff1b*GFP-expressing steroidogenic tissue (ROI marked by orange lines) is normalized to the size of the cluster, with the number of embryos indicated in parentheses. The difference between 2-dpf control group and any of the other groups was analyzed by Student's t-test. *P<0.05, N.S., not significant. A, anterior; P, posterior; L, left; R, right; D, dorsal; V, ventral. Broken yellow lines indicate position of the midline. Abbreviations: notochord (NC). Scale bar, 25 µm.

The interaction of cancerous epithelial cells with Fn *in vitro* promotes EMT [53], [54]. The morphology of the interrenal steroidogenic tissue and the associated Fn-enriched microenvironment suggested that an EMT-like phenotypic change could occur during interrenal medial extension. An examination of interrenal tissue morphology by high resolution Nomarski microscopy revealed that while steroidogenic cells positive for 3β-Hsd activity formed a cluster to the right of the midline by 48 hpf (Figure 6A, B), protrusions were detected at 48 hpf that became more evident by 60 hpf (Figure 6C) and continuously spread across the midline until a bilobed structure was formed by 84 hpf (Figure 6E). During the morphogenetic movement, interrenal steroidogenic cells became more loosely associated with each other and demonstrated a mesenchymal-like phenotype with cell surface protrusions (Figure 6D, E). These morphological features likely reflected an EMT-like process during organ assembly.

To confirm whether interrenal steroidogenic cells undergo a transformation from epithelial to mesenchymal phenotypes, the expression of β-Catenin—a marker for adherent junctions in epithelial cells [55]—was examined. At 2 dpf, β-Catenin was clearly detected at cell-cell junctions of interrenal steroidogenic cells visible by GFP expression in *ff1bEx2: GFP* embryos (Figure 6F–F'), while at 2.5 dpf, β-Catenin was markedly reduced within the steroidogenic tissue cluster (Figure 6G–G'), reflecting the adoption of a mesenchymal-like character.

Since an EMT-like change occurred in steroidogenic cells during interrenal medial extension, the roles of hemodynamic forces and Fn-pFak signaling in this process was assessed. The accumulation of β-Catenin was observed in the interrenal tissue of blood flow-deficient embryos generated by *tnnt2a*MO microinjection (Figure 6H–H') or 6 mM 2,3-BDM treatment (Figure 6I–I'), providing evidence that blood flow induces an EMT-like change in steroidogenic cells. Since blood flow regulates interrenal medial migration via Fn-pFak signaling (Figure 4), Fn signaling was inhibited without perturbing blood flow to determine whether the EMT-like change in steroidogenic cells could be repressed. The RGD peptide, an antagonist of Fn, was applied to *Tg(ff1bEx2: GFP)* embryos at a concentration of 100 µM starting from 26 hpf, when circulation is initiated; in these embryos, junctional β-Catenin distribution was significantly higher than in controls at 2.5 dpf (Figure 6J–J', M). RGD effectively reduced pFak level in the interrenal area (Figure S5), thus suggesting that the inhibition of pFak signaling was responsible for the observed suppression of EMT-like changes in steroidogenic cells. Moreover, β-Catenin accumulation at cell-cell junctions was evident in both *klf2a* and *mmp2* morphants at 2.5 dpf (Figure 6K–K', L–L', M) as compared to the control embryo.

Consistent with the results of β-Catenin expression, immunohistochemial analysis of E-cadherin, a central component of cell-cell adhesion junction which is required for the formation of epithelia [56], [57], detected a clear epithelial phenotype of the interrenal tissue at 2 dpf (Figure S6A, A'). At 2.5 dpf, the E-cadherin expression was reduced at the interrenal tissue where a migratory phenotype was manifested (Figure S6B, B'). In contrast, junctional E-cadherin distribution at the interrenal tissue was not reduced in 2.5 dpf embryos where interrenal medial migration was suppressed by a disruption of either circulation (Figure S6C, C') or pFak-mediated signaling (Figure S6D, D'), or hemodynamics-regulated molecules (Figure S6E, E', F, F'). It was noted that RGD-treated embryos and *mmp2* morphants at 2.5 dpf displayed a higher expression level of junctional E-cadherin than control embryos at 2 dpf did (Figure S6G), with the underlying mechanism remaining unclear. Nevertheless, the immunohistochemistry results of β-Catenin and E-cadherin both indicated a clear reduction of epithelial nature at the interrenal tissue from 2 to 2.5 dpf. Moreover, mesenchymal phenotype of the interrenal tissue at 2.5 dpf correlated well with a rise of Vimentin expression in the *ff1b*-expressing steroidogenic cells (Figure S7A, A', B, B'). Vimentin, a type III intermediate filament protein and a widely used mesenchymal marker, plays a predominant role for inducing changes in cell shape, adhesion and motility during the EMT [58], [59]. In contrast to the reduction of β-Catenin and E-cadherin during interrenal medial extension, the Vimentin expresison is significantly increased from 2 to 2.5 dpf, and this accumulation of Vimentin was not detected in embryos deficient in either blood flow (Figure S7C, C') or pFak signaling (Figure S6D, D'), or hemodynamic transducers (Figure S7E, E', F, F'). Therefore, an inverse correlation between epithelial and mesenchymal markers was observed during interrenal medial expression. Taken together, these results indicate that blood flow, through mechanotransduction and Fn-pFak signaling, promotes EMT-like changes in steroidogenic cells during interrenal organ assembly.

### Migration of Differentiated Chromaffin Cells Requires Blood Flow but is Independent of Mechanotransduction and Fn-Mediated Signaling

**Figure 7:**
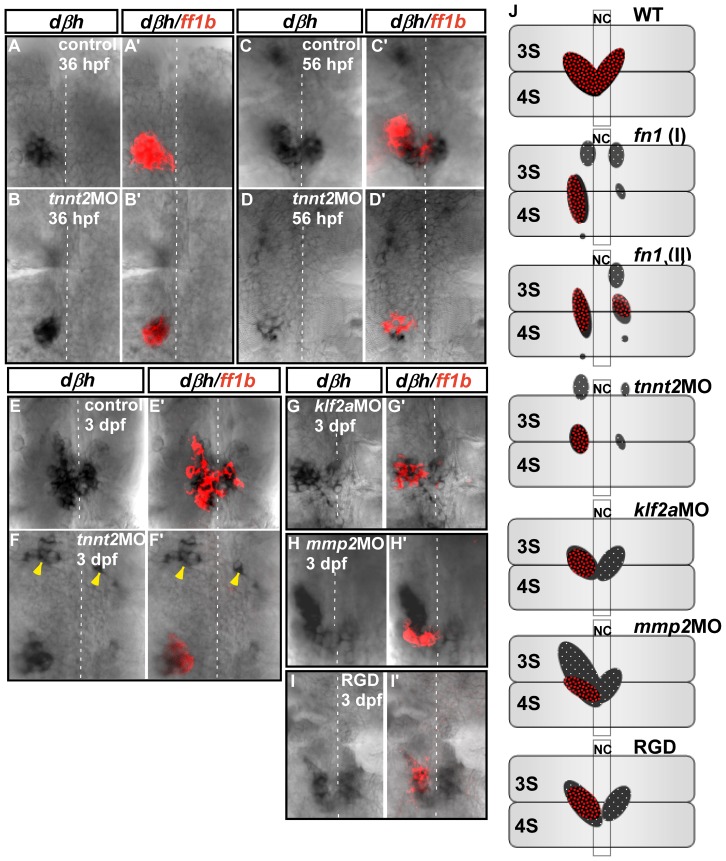
Interaction between interrenal steroidogenic and chromaffin cells in *tnnt2a*, *klf2a*, and *mmp2* morphants and RGD-treated embryos during interrenal gland assembly. Double ISH assays showing colocalization of *ff1b* (red) and *dbh* (black) transcripts in uninjected control embryos and *tnnt2a* morphants at (A, A'; B, B') 36 hpf (n = 3 and 6, respectively), (C, C'; D, D') 56 hpf (n = 3 and 5, respectively), and (E, E'; F, F') 3 dpf (n = 17 and 15, respectively), and in (G, G') *klf2a* (n = 9) and (H, H') *mmp2* (n = 8) morphants and (I, I') RGD-treated embryos (n = 18) at 3 dpf. Ventral flat mount views are shown for representative embryos in each group, oriented with anterior at the top. Yellow arrowheads indicate chromaffin cell clusters that failed to converge at the interrenal area in *tnnt2a* morphants. Broken white lines indicate position of the midline. (J) Schematic representation of various phenotypic defects associated with interrenal organ assembly. Panels show ventral views of wild-type, mutant, morphant, and drug-treated embryos at 3 dpf, oriented with anterior at the top. Phenotypes depicted for *cloche* (*clo*) and *fn1* mutants are based on previous reports [9], [21]. Abbreviations: notochord (NC), the third somite (3S), the fourth somite (4S).

To determine whether blood flow also regulates the development of the chromaffin cell lineage, ISH was performed to detect transcript expression of *ff1b* and *dβh*, markers for steroidogenic and chromaffin cell lineages, respectively (Figure 7). Consistent with the findings of our earlier study [19], the integration of the two cell populations was detected as early as 36 hpf (Figure 7A–A'), and was not affected in *tnnt2a* morphants (Figure 7B–B'), while interrenal medial migration and organ assembly were observed at 56 hpf and 3 dpf (Figure 7C–C', E–E'), respectively. Since the medial extension of steroidogenic tissue was inhibited in *tnnt2a* morphants, chromaffin cells that reached the interrenal region remained closely associated with steroidogenic cells, and were located to the right of the midline (Figure 7D–D', F–F'). While the convergence of differentiated chromaffin cells colonizing the interrenal organ was completed by 3 dpf in wild-type embryos (Figure 7E'), clusters of differentiated chromaffin cells were located outside the interrenal region in *tnnt2a* morphants (Figure 7F–F'), apparently due to the unsuccessful migration of chromaffin cells, indicating that although they are still capable of interacting with steroidogenic cells, their migration is defective in blood flow-deficient embryos, leading to an incomplete assembly of the interrenal organ.

Interestingly, while blood flow was required, mechanotransduction and Fn-pFak signaling were dispensable for chromaffin cell migration. Embryos injected with *klf2a*MO or *mmp2*MO, or treated with 100 µM RGD starting from 26 hpf, had defective medial migration of *ff1b-*expressing cells but normal convergence of chromaffin cells at the midline (Figure 7G–I, G'–I'), resulting in only partial integration of the two cell lineages. This implies that the migration of steroidogenic and chromaffin cells are differentially modulated by blood flow.

## Discussion

**Figure 8:**
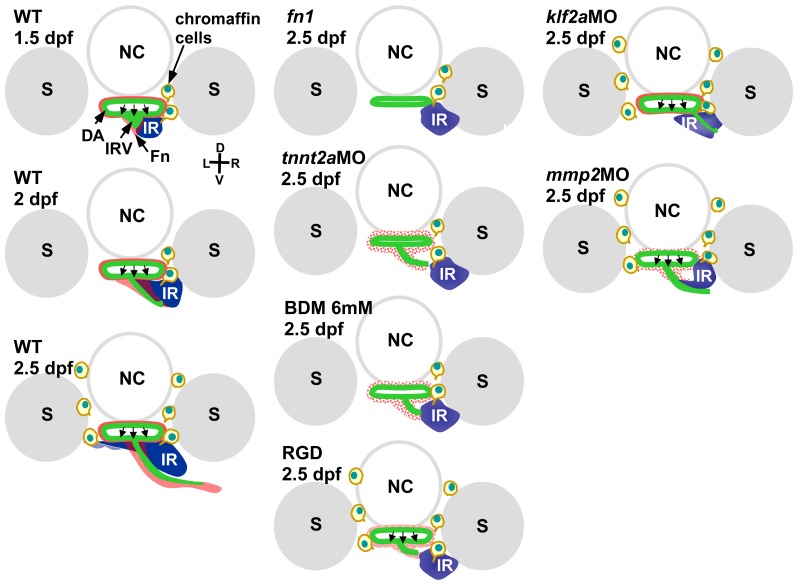
Schematic representation of interrenal steroidogenic tissue (IR) and chromaffin cell integration that takes place in the vicinity of the DA and extending IRV. An Fn-enriched microenvironment promotes medial extension of the steroidogenic tissue and culminates in steroidogenic-chromaffin interactions on both sides of the midline (left); various defects in extension lead to incomplete assembly of the interrenal organ (middle and right). D, dorsal; V, ventral; L, left; R, right. Abbreviations: notochord (NC). Somite (S).

The results of this study indicate that in addition to supplying steroids and maintaining tissue homeostasis, blood flow ensures maximal interaction between steroidogenic and chromaffin cells in teleosts (Figure 8). During interrenal organ assembly, hemodynamic forces pattern the vascular microenvironment and regulate the morphology of steroidogenic cells and the associated angiogenic endothelium. The data presented here illustrate a mechanism by which an EMT-like process and tissue-tissue interactions can be modulated by blood flow. A disruption of the vascular microenvironment or mechanotransduction perturbs steroidogenic tissue morphogenesis but not chromaffin cell migration, indicating that blood flow regulates these processes through different pathways.

The obstruction of blood flow in the zebrafish embryo produced a phenotype similar to that of the *fn1* mutant [19] (Figure 7), providing evidence that the interrenal Fn-enriched microenvironment is regulated by hemodynamic forces (Figure 4). However, while blood flow-deficient embryos have similar defects in steroidogenic and chromaffin cell migration, 20%–30% of *fn1* mutants exhibit a more severe phenotype, where the bilateral fusion of early interrenal tissues is unsuccessful (Figure 7). The variable expressivity of this phenotype in *fn1* mutants could be due to a more profound effect of Fn deficiency on early development prior to the onset of blood flow [41], [60]. Indeed, early bilateral interrenal tissues arise in close association with pre-vascular angioblasts, and their fusion occurs in parallel with the assembly of axial vasculature independently of the initiation of blood flow [9], [52].

The phenotype of the RGD-treated embryo—that is, defective migration of steroidogenic but not chromaffin cells—was different from that of the *fn1* mutant, in which the migration of both cell types was compromised (Figs. 7 and 8). Fn regulates the fusion of bilateral cardiac primordia, and is therefore essential for the development of myocardial epithelia [41]; thus, *fn1* mutants have impaired cardiac function and consequently, reduced blood flow, which may cause the aberrant migration of chromaffin cells. Nevertheless, these cell clusters in *fn1* mutants were more dispersed than in *tnnt2a* morphants (Figure 7J), suggesting that the presence of Fn ensures that the microenvironment stimulates chromaffin cell migration prior to the onset of circulation. Trunk neural crest cells, from which the sympathochromaffin lineage is derived, migrate along the medial surface of each somite but not the somite boundary where Fn accumulates [60], [61]. However, disrupted somite formation in the *fn1* mutant leads to the uncoupling of slow- and fast-twitch muscle fibers, and hence a disorganized myofibril pattern [62], which is another factor that could compromise chromaffin cell migration. In contrast, the RGD-treatment in this study was initiated at 26 hpf without evident perturbation of cardiac flow and somite morphology, which might explain why chomaffin cells migrate normally in RGD-treated embryos.

EMT is initiated by transforming, fibroblast, epithelial, and hepatocyte growth factors as well as the oncogene Harvey rat sarcoma. During this process, there is a downregulation of epithelial and concomitant upregulation of mesenchymal markers, while cells assume a proliferative and migratory character [35]. EMT is similar to the endothelial-to-mesenchymal transition (EnMT), a critical step in vertebrate heart development in which endothelial endocardial cells give rise to heart cushion cells that form the mesenchymal portion of septa and valves [63]. In various vertebrate models including zebrafish, alterations in hemodynamic forces during cardiogenesis have profound effects on cardiac septation and valvulogenesis [3], [4], [46], [64], suggesting that EnMT is regulated by hemodynamics. The present study underscores a novel role for hemodynamics in the EMT-like behavior of vessel-associated endocrine cells via modulation of the vascular microenvironment and Fn-pFak signaling.

In addition to being an epithelial cell marker, β-Catenin also transduces canonical Wnt signals and is implicated in cell proliferation [65]. Cytoplasmic β-Catenin is stabilized upon activation of Wnt signaling, leading to its translocation to the nucleus, where it interacts with T-cell factors (TCFs) to stimulate the transcription of target genes. There was no obvious enrichment of nuclear β-Catenin detected in steroidogenic cells during interrenal organ assembly (Figure 6), and it is possible that canonical Wnt signaling is not involved in zebrafish interrenal development. Nevertheless, Wnt4 can inhibit β-Catenin/TCF signaling by redirecting nuclear β-Catenin to the membrane [66]. During mammalian sex differentiation, Wnt4 inhibits the migration of endothelial and steroidogenic cells into the female gonad [67], and is also expressed in the outermost region of the adrenal cortex, where it may play a role in migration events that segregate adrenal and gonadal lineages during early development [68]. It therefore remains to be explored whether Wnt signals are directly or indirectly regulated by hemodynamic forces and thereby guide cell migration during interrenal organ assembly.

It will be of interest to explore whether other morphogens participate in the interrenal organogenesis. Pregnenolone, a steroid produced from cholesterol by the steroidogenic enzyme Cyp11a1, promotes cell migration by activating CLIP-170 and stabilizing microtubules [69], [70]. The function of pregnenolone for cell movement has been well studied in the zebrafish gastrulation which is well before the onset of blood flow as well as interrenal organogenesis. While *cyp11a1* is expressed at the zebrafish interrenal tissue by 1 dpf [18], it remains unclear whether pregnelolone would also form a morphogen gradient during the interrenal morphogenetic movement. On the other hand, the molecular and cellular mechanisms by which blood flow regulates chromaffin cell migration remain to be explored. In the blood flow-deficient zebrafish embryo, the expression of endothelial CXCR4a is upregulated during collateral formation [71]. CXCR4a is the G protein-coupled receptor for the chemokine stromal cell-derived factor 1 (SDF-1). SDF-1, along with Bone Morphogenetic Proteins (BMPs) and Neuregulin 1 of the epidermal growth factor family, are the three groups of paracrine factors that participate in the generation of sympatho-adrenal progenitors from the neural crest [12], [72], [73], [74]. BMPs are produced by the DA and are critical for the production of SDF-1 and Neuregulin 1 in the vicinity of the DA, while SDF-1 and Neuregulin 1 function as chemoattractants for the migration of neural crest cells [15]. It will therefore be intriguing to examine whether a loss of blood flow would influence the activity of SDF-1 through upregulating CXCR4a expression.

## Supporting Information

Figure S1 **Effect of norepinephrine on the heart beat of zebrafish.** Norepinephrine treatments resulted in a dose-dependent increase of heart rate at 33 hpf. The difference between groups treated with various concentrations of norepinephrine was analyzed by Student's t-test. *P<0.05, ***P<0.0005.
(TIF)

Figure S2 **Interrenal cell migration suppressed by 2,3-BDM was recovered following the removal of 2,3-BDM.** The interrenl tissue stained by 3β-Hsd activity assay in the control *Tg(kdrl: GFP)^s843^* embryo continued to extend across the midline from 2.5 dpf (A-A'') to 3 dpf (C-C''), while migration of interrenal cells was repressed by 2,3-BDM treatment at 6 mM from 1.5 to 2.5 dpf (B-B'') or 3 dpf (D-D''). Migration of interrenal cells was recovered at 3 dpf as 2,3-BDM was washed out at 2.5 dpf (E-E''). Protrusions of extending interrenal tissues (red arrows) were detected in control as well as recovered embryos. Broken white lines indicate position of the midline. Abbreviations: glomerulus (G), posterior cardinal vein (PCV). Scale bar, 50 µm.
(TIF)

Figure S3 **Effects of blood flow inhibition on IRV growth was reversible following the removal of 2,3-BDM.** The IRV in the control *Tg(kdrl: GFP)^s843^* embryo continued to extend from 2.5 dpf (A, A') to 3 dpf (C, C'), while the IRV growth was repressed by 2,3-BDM treatment at 6 mM from 1.5 to 2.5 dpf (B, B') or 3 dpf (D, D'). Extension of IRV was recovered at 3 dpf as 2,3-BDM was washed out at 2.5 dpf (E, E'). The interrenal tissue (IR) was detected by 3β-Hsd acitivity assay. D, dorsal; V, ventral; L, left; R, right. Broken yellow lines indicate position of the midline. Abbreviations: notochord (NC), somite (S). Scale bar, 50 µm.
(TIF)

Figure S4 **Effect of l-NAME on morphogenetic movements of interrenal steroidogenic tissue and IRV formation.** (A–A'') *Tg(kdrl: GFP)^s843^* embryos treated with 100 µM l-NAME from 1.5 dpf onwards had interrenal steroidogenic tissue (IR) morphology (orange arrows) and (B–B'') IRV length similar to control embryos (Figure 3A, A', D) at 2.5 dpf (n = 8). The activity of endothelial NO synthase was inhibited by l-NAME at concentrations higher than 10 µM [50]. D, dorsal; V, ventral; L, left; R, right. Abbreviations: posterior cardinal vein (PCV). Scale bar, 50 µm.
(TIF)

Figure S5 **Effect of RGD treatment on pFAK distribution in the interrenal region.** Transverse sections of *Tg(kdrl: GFP)^s843^* embryos either untreated (control; n = 6) or treated with 100 µM RGD peptide (n = 10) from 26 hpf and harvested at 2.5 dpf for evaluation of 3β-Hsd activity and pFak level by IHC. Sections are shown of a representative embryo from each group, oriented with the dorsal side at the top. Abbreviations: interrenal tissue (IR), notochord (NC), somite (S).
(TIF)

Figure S6 **Steroidogenic cells display a decrease of E-cadherin expression which is induced by hemodynamic forces and pFak signaling.** E-cadherin in the fluorescent steroidogenic tissue of *Tg(ff1bEx2: GFP)* embryos was decreased from 2 dpf (A-A') to 2.5 dpf (B-B'). The decrease in E-cadherin was not observed in (C, C') *tnnt2a* morphants, (D, D') RGD-treated embryos, or (E, E') *klf2a* or (F, F') *mmp2* morphants. Sections are shown of a representative embryo from each treatment group. (F) Fluorescence intensity of E-cadherin in *ff1b*GFP-expressing steroidogenic tissue is normalized to the size of the cluster, with the number of embryos indicated in parentheses. The difference between 2-dpf control group and any of the other groups was analyzed by Student's t-test. *P<0.05, **P<0.005, N.S., not significant. D, dorsal; V, ventral; L, left; R, right. Abbreviations: notochord (NC). Scale bar, 25 µm.
(TIF)

Figure S7 **Steroidogenic cells display a rise of Vimentin expression which is induced by hemodynamic forces and pFak signaling.** Vimentin in the steroidogenic tissue (marked by green fluorescence) of *Tg(ff1bEx2: GFP)* embryos was increased from 2 dpf (A-A') to 2.5 dpf (B-B'). The increase in Vimentin was not observed in (C, C') *tnnt2a* morphants, (D, D') RGD-treated embryos, or (E, E') *klf2a* or (F, F') *mmp2* morphants. Sections are shown of a representative embryo from each treatment group. (G) Fluorescence intensity of Vimentin in *ff1b*GFP-expressing steroidogenic tissue (ROI marked by orange lines) is normalized to the size of the cluster, with the number of embryos indicated in parentheses. The difference between 2-dpf control group and any of the other groups was analyzed by Student's t-test. **P<0.005, N.S., not significant. D, dorsal; V, ventral; L, left; R, right. Abbreviations: notochord (NC). Scale bar, 25 µm.
(TIF)

Video S1 **Complete blockage of blood flow in the posterior cardinal vein of a 36-hpf embryo treated with 6 mM 2,3-BDM.**
(WMV)

Video S2 **Normal blood flow in the posterior cardinal vein of a wild-type 36-hpf embryo.**
(WMV)

Video S3 **Partial reduction in blood flow in the posterior cardinal vein of a 36-hpf embryo treated with 2 mM 2,3-BDM.**
(WMV)
