# Supplementary figures and images for: The Hemodynamically-Regulated Vascular Microenvironment Promotes Migration of the Steroidogenic Tissue during Its Interaction with Chromaffin Cells in the Zebrafish Embryo
Source: PLoS One. 2014 Sep 23;9(9):e107997. doi: 10.1371/journal.pone.0107997 (PMC4172588; doi:10.1371/journal.pone.0107997)

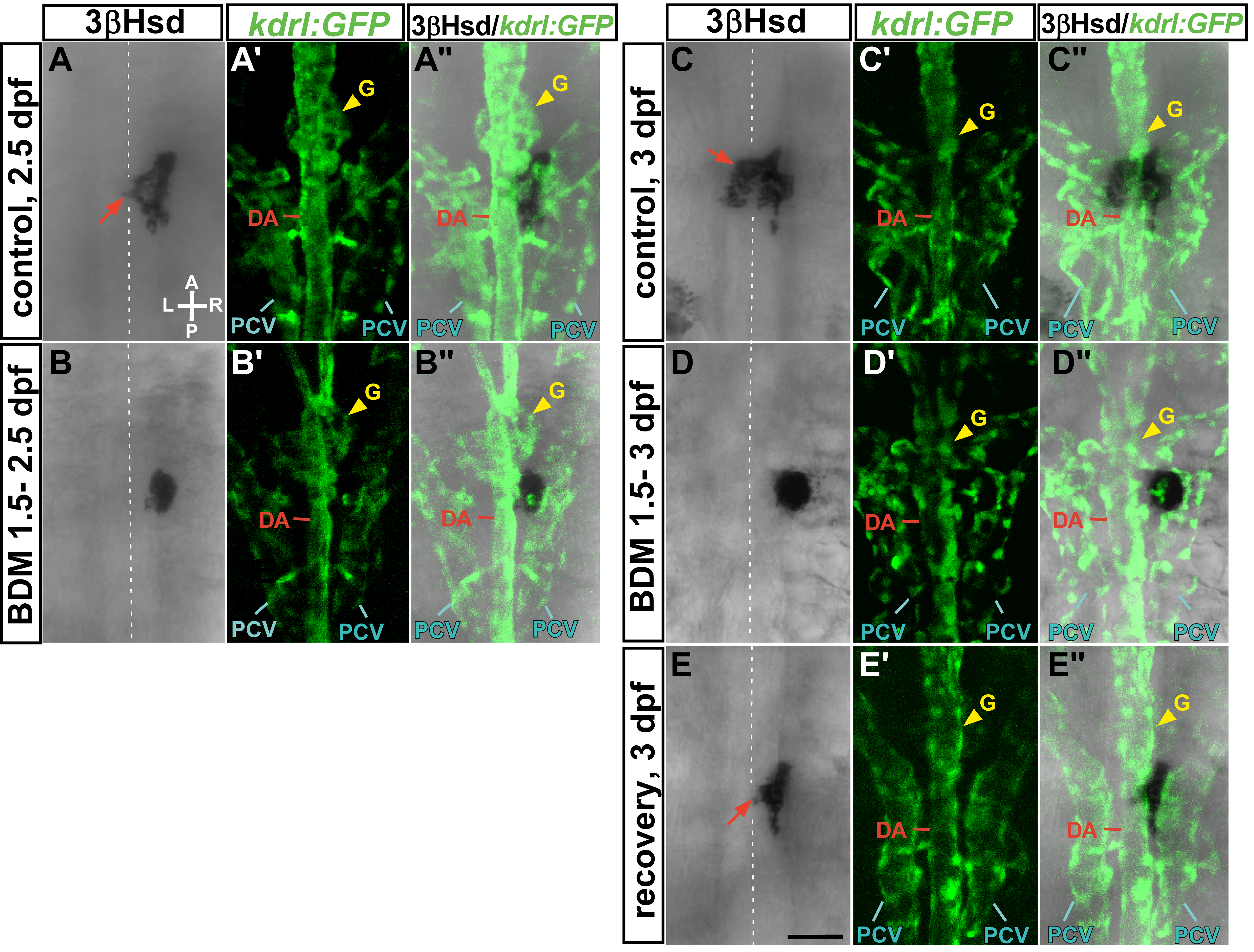

Supplement: Figure S2 — Interrenal cell migration suppressed by 2,3-BDM was recovered following the removal of 2,3-BDM. The interrenl tissue stained by 3β-Hsd activity assay in the control Tg(kdrl: GFP)s843 embryo continued to extend across the midline from 2.5 dpf (A-A'') to 3 dpf (C-C''), while migration of interrenal cells was repressed by 2,3-BDM treatment at 6 mM from 1.5 to 2.5 dpf (B-B'') or 3 dpf (D-D''). Migration of interrenal cells was recovered at 3 dpf as 2,3-BDM was washed out at 2.5 dpf (E-E''). Protrusions of extending interrenal tissues (red arrows) were detected in control as well as recovered embryos. Broken white lines indicate position of the midline. Abbreviations: glomerulus (G), posterior cardinal vein (PCV). Scale bar, 50 µm. (TIF) [file pone.0107997.s002.tif]

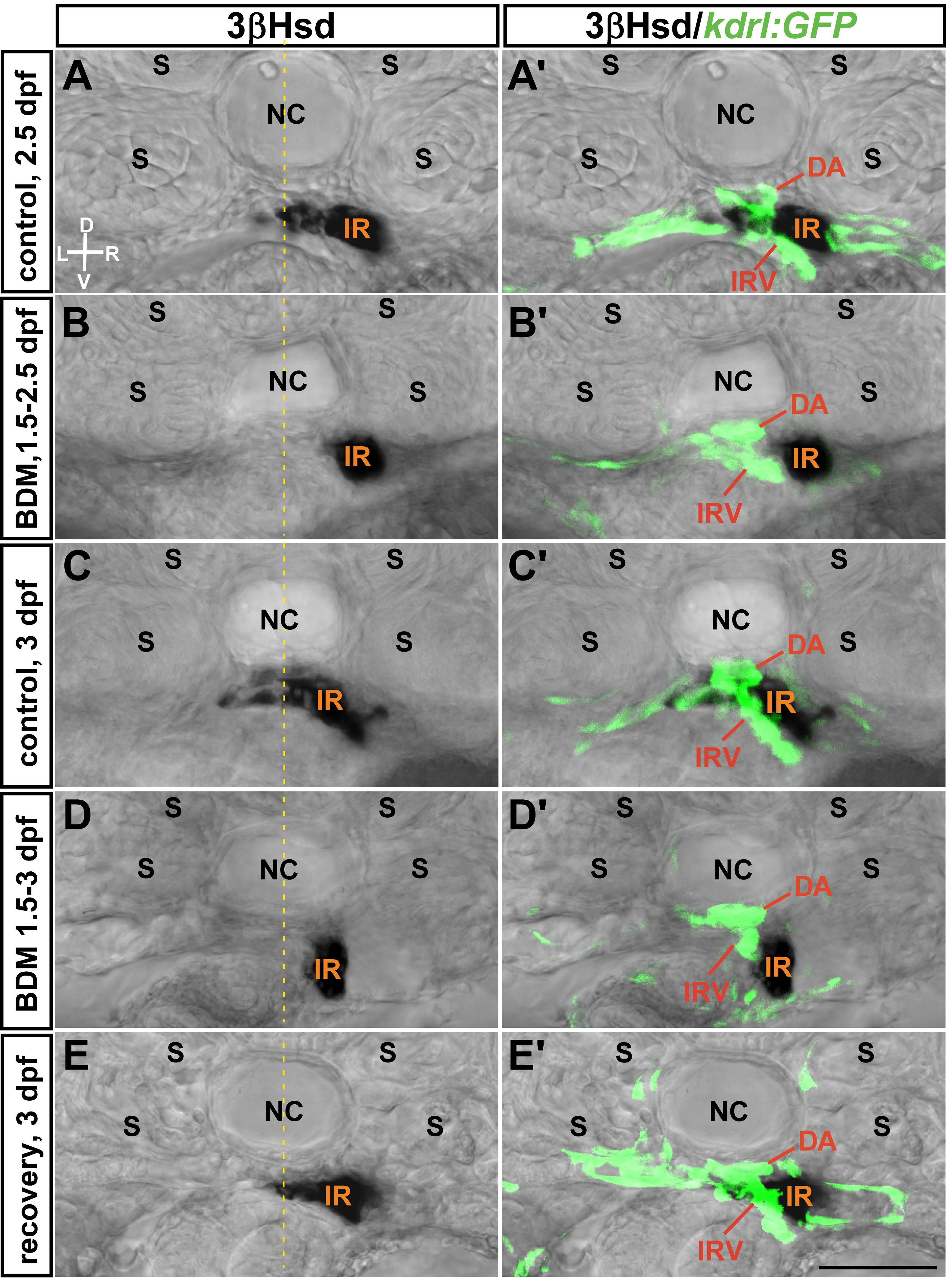

Supplement: Figure S3 — Effects of blood flow inhibition on IRV growth was reversible following the removal of 2,3-BDM. The IRV in the control Tg(kdrl: GFP)s843 embryo continued to extend from 2.5 dpf (A, A') to 3 dpf (C, C'), while the IRV growth was repressed by 2,3-BDM treatment at 6 mM from 1.5 to 2.5 dpf (B, B') or 3 dpf (D, D'). Extension of IRV was recovered at 3 dpf as 2,3-BDM was washed out at 2.5 dpf (E, E'). The interrenal tissue (IR) was detected by 3β-Hsd acitivity assay. D, dorsal; V, ventral; L, left; R, right. Broken yellow lines indicate position of the midline. Abbreviations: notochord (NC), somite (S). Scale bar, 50 µm. (TIF) [file pone.0107997.s003.tif]

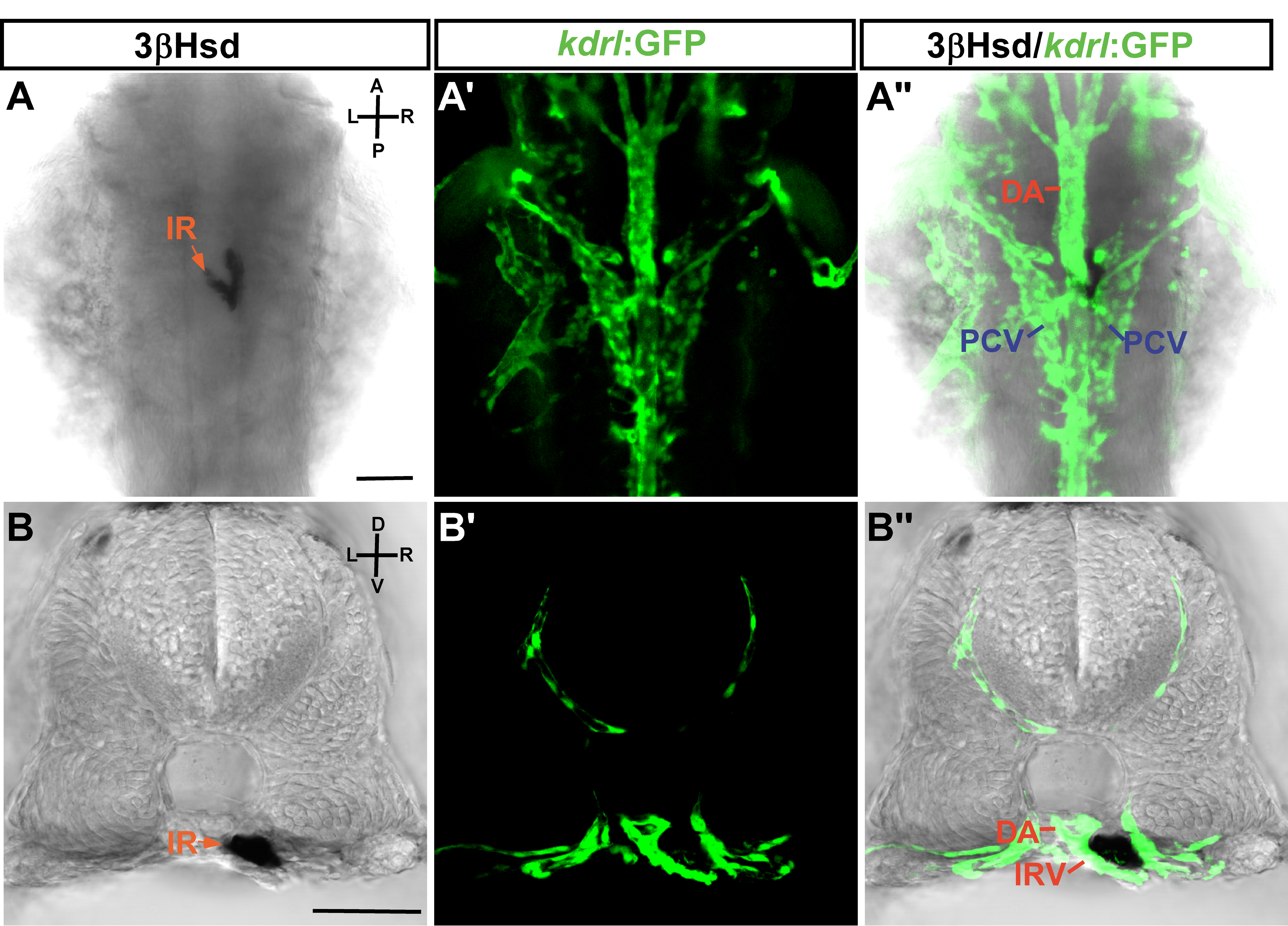

Supplement: Figure S4 — Effect of l-NAME on morphogenetic movements of interrenal steroidogenic tissue and IRV formation. (A–A'') Tg(kdrl: GFP)s843 embryos treated with 100 µM l-NAME from 1.5 dpf onwards had interrenal steroidogenic tissue (IR) morphology (orange arrows) and (B–B'') IRV length similar to control embryos (Figure 3A, A', D) at 2.5 dpf (n = 8). The activity of endothelial NO synthase was inhibited by l-NAME at concentrations higher than 10 µM [50]. D, dorsal; V, ventral; L, left; R, right. Abbreviations: posterior cardinal vein (PCV). Scale bar, 50 µm. (TIF) [file pone.0107997.s004.tif]

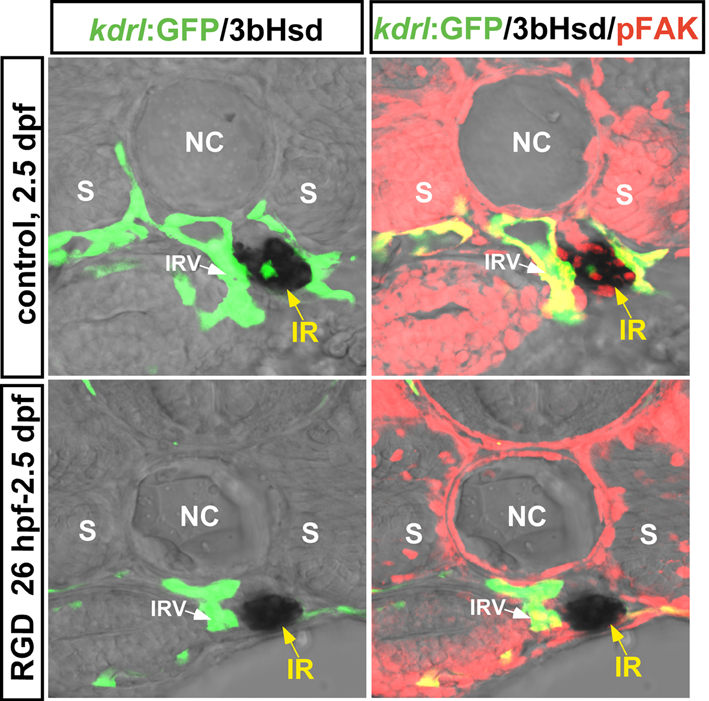

Supplement: Figure S5 — Effect of RGD treatment on pFAK distribution in the interrenal region. Transverse sections of Tg(kdrl: GFP)s843 embryos either untreated (control; n = 6) or treated with 100 µM RGD peptide (n = 10) from 26 hpf and harvested at 2.5 dpf for evaluation of 3β-Hsd activity and pFak level by IHC. Sections are shown of a representative embryo from each group, oriented with the dorsal side at the top. Abbreviations: interrenal tissue (IR), notochord (NC), somite (S). (TIF) [file pone.0107997.s005.tif]

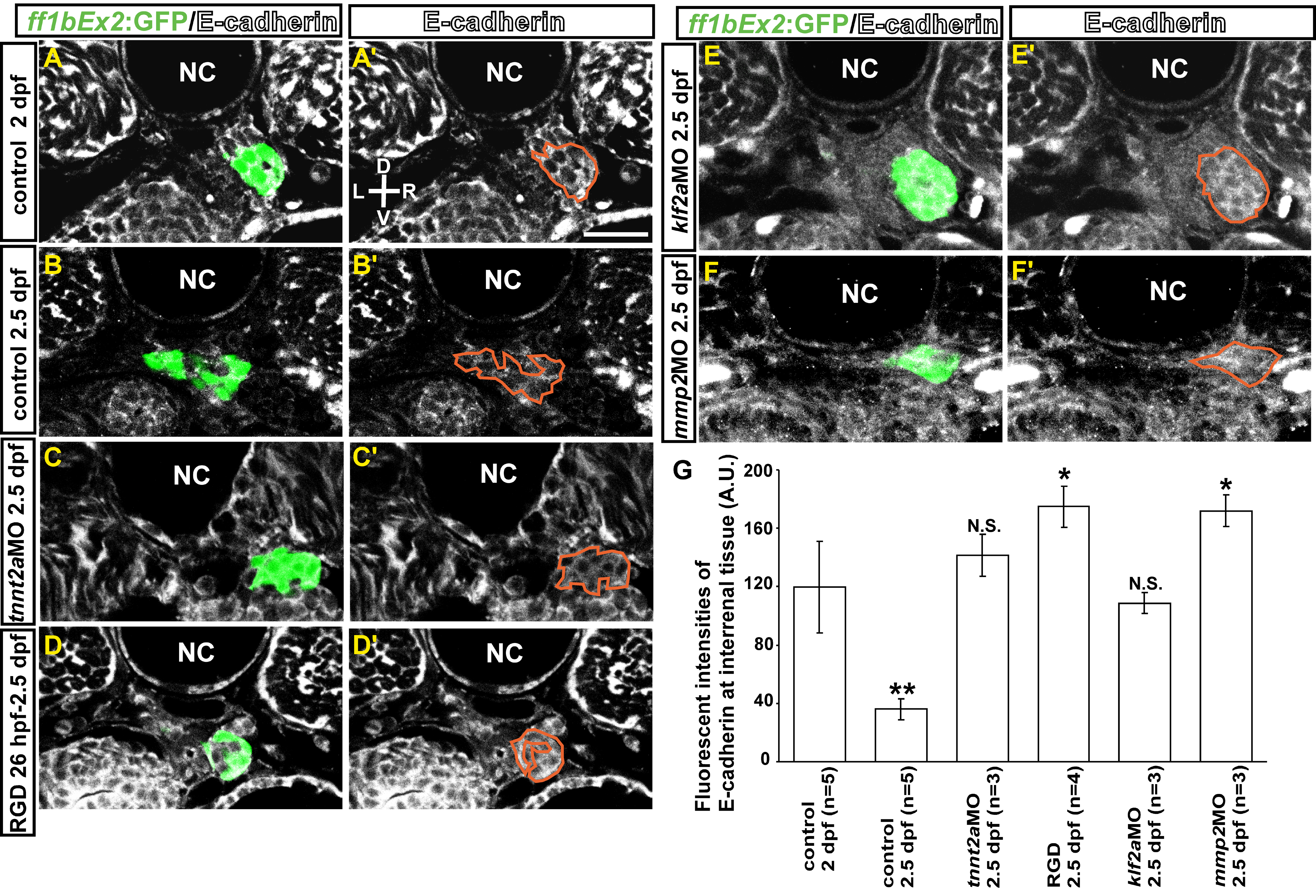

Supplement: Figure S6 — Steroidogenic cells display a decrease of E-cadherin expression which is induced by hemodynamic forces and pFak signaling. E-cadherin in the fluorescent steroidogenic tissue of Tg(ff1bEx2: GFP) embryos was decreased from 2 dpf (A-A') to 2.5 dpf (B-B'). The decrease in E-cadherin was not observed in (C, C') tnnt2a morphants, (D, D') RGD-treated embryos, or (E, E') klf2a or (F, F') mmp2 morphants. Sections are shown of a representative embryo from each treatment group. (F) Fluorescence intensity of E-cadherin in ff1bGFP-expressing steroidogenic tissue is normalized to the size of the cluster, with the number of embryos indicated in parentheses. The difference between 2-dpf control group and any of the other groups was analyzed by Student's t-test. *P<0.05, **P<0.005, N.S., not significant. D, dorsal; V, ventral; L, left; R, right. Abbreviations: notochord (NC). Scale bar, 25 µm. (TIF) [file pone.0107997.s006.tif]

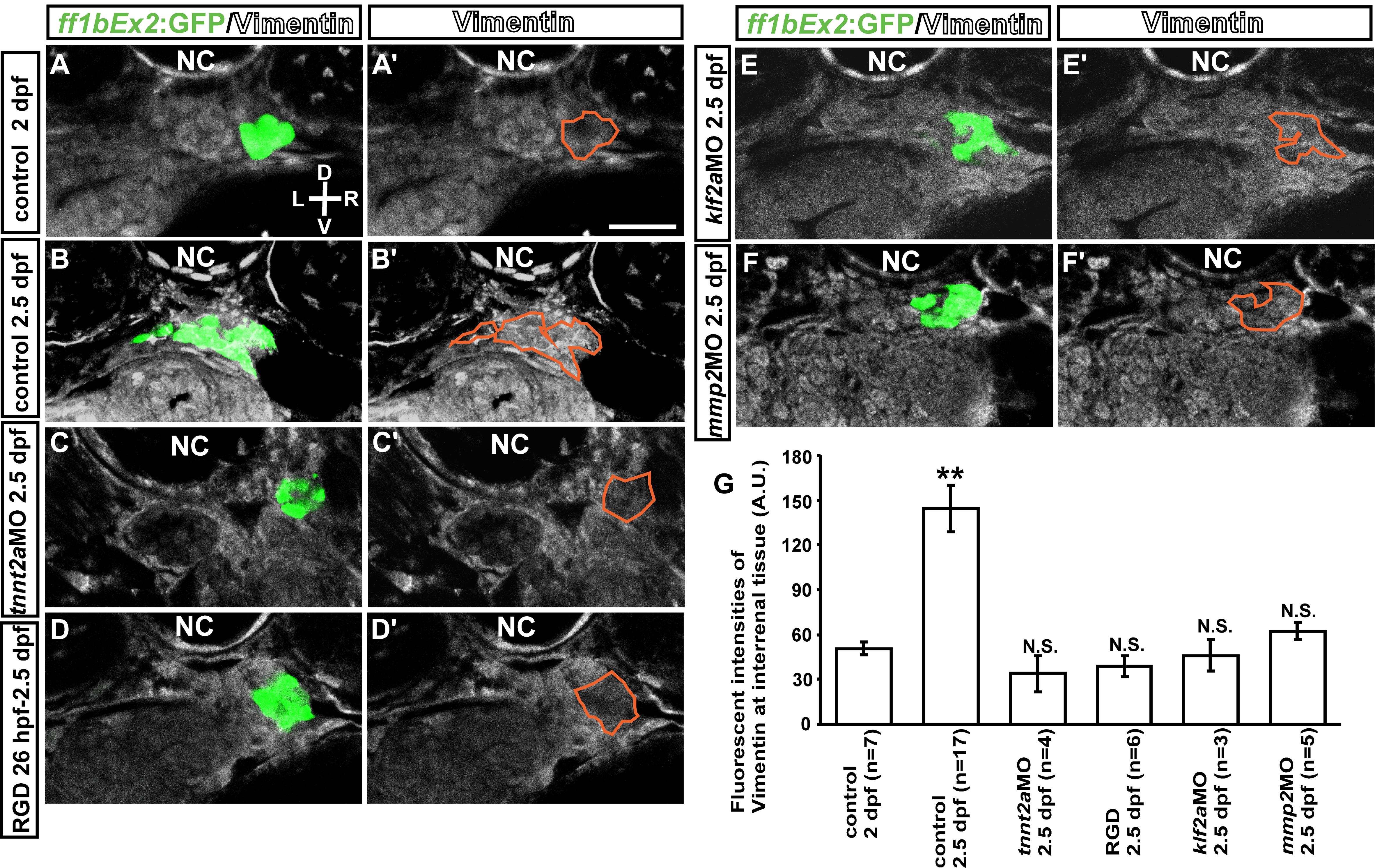

Supplement: Figure S7 — Steroidogenic cells display a rise of Vimentin expression which is induced by hemodynamic forces and pFak signaling. Vimentin in the steroidogenic tissue (marked by green fluorescence) of Tg(ff1bEx2: GFP) embryos was increased from 2 dpf (A-A') to 2.5 dpf (B-B'). The increase in Vimentin was not observed in (C, C') tnnt2a morphants, (D, D') RGD-treated embryos, or (E, E') klf2a or (F, F') mmp2 morphants. Sections are shown of a representative embryo from each treatment group. (G) Fluorescence intensity of Vimentin in ff1bGFP-expressing steroidogenic tissue (ROI marked by orange lines) is normalized to the size of the cluster, with the number of embryos indicated in parentheses. The difference between 2-dpf control group and any of the other groups was analyzed by Student's t-test. **P<0.005, N.S., not significant. D, dorsal; V, ventral; L, left; R, right. Abbreviations: notochord (NC). Scale bar, 25 µm. (TIF) [file pone.0107997.s007.tif]
